# Three-Dimensional Microstructural Imaging of Sulfur Poisoning-Induced Degradation in a Ni-YSZ Anode of Solid Oxide Fuel Cells

**DOI:** 10.1038/srep05246

**Published:** 2014-06-10

**Authors:** William M. Harris, Jeffrey J. Lombardo, George J. Nelson, Barry Lai, Steve Wang, Joan Vila-Comamala, Mingfei Liu, Meilin Liu, Wilson K. S. Chiu

**Affiliations:** 1 HeteroFoaM Center, a DOE Energy Frontier Research Center; 2 Department of Mechanical Engineering, University of Connecticut; 3 Advanced Photon Source, Argonne National Laboratory; 4 School of Materials Science and Engineering, Georgia Institute of Technology

## Abstract

Following exposure to ppm-level hydrogen sulfide at elevated temperatures, a section of a solid oxide fuel cell (SOFC) Ni-YSZ anode was examined using a combination of synchrotron-based x-ray nanotomography and x-ray fluorescence techniques. While fluorescence measurements provided elemental identification and coarse spatial mapping, x-ray nanotomography was used to map the detailed 3-D spatial distribution of Ni, YSZ, and a nickel-sulfur poisoning phase. The nickel-sulfur layer was found to form a scale covering most of the exposed nickel surface, blocking most fuel reformation and hydrogen oxidation reaction sites. Although the exposure conditions precluded the ability to develop a detailed kinetic description of the nickel-sulfur phase formation, the results provide strong evidence of the detrimental effects of 100 ppm hydrogen sulfide on typical Ni-YSZ anode materials.

Solid oxide fuel cells (SOFC) are a promising power source due to their excellent fuel flexibility and high energy efficiency at low cost. However, the conventional anode material, a composite consisting of nickel and yttria-stabilized zirconia (Ni-YSZ) is highly susceptible to poisoning-induced degradation when the fuel stream contains small amounts of impurities such as hydrogen sulfide H_2_S^1^,^2^,^3^. While a number of alternative anode materials have been proposed to alleviate this problem, Ni-YSZ still remains a common SOFC anode due to its excellent physical, chemical, and thermal-mechanical compatibility with other SOFC components^4^,^5^,^6^,^7^,^8^. Understanding the sulfur poisoning process in a Ni-YSZ anode is critical to mitigating the sulfur poisoning effect on the electrochemical activity of the Ni-YSZ towards fuel oxidation.

Experimental observations frequently report an immediate, dramatic drop in cell performance followed by a slower, longer decrease occurring with extended exposure to ppm-level H_2_S in hydrogen H_2_. This behavior is described in terms of two distinct poisoning processes^9^,^10^,^11^,^12^,^13^. Upon immediate H_2_S exposure, elementary S is adsorbed on the Ni surface, which blocks the active sites for the hydrogen oxidation reaction^14^,^15^. A slower process, the sulfidation of Ni to produce various bulk nickel-sulfur compounds, results in longer term performance degradation due to irreversible alterations of the microstructure of the anode material^16^.

However, it is still not quite clear at this point under what conditions (temperature, time, and H_2_S concentration) each of the two poisoning regimes is applicable due to a lack of understanding about the fundamental mechanisms. A numerically-generated phase diagram produced by Wang et al. using density functional theory (DFT) has suggested that under typical SOFC operating conditions with ppm-level H_2_S, surface-adsorbed S is more likely than bulk nickel-sulfide formation^17^. However, operation under higher H_2_S concentration or lower temperature (such as during cell start-up or shut-down) could produce conditions suitable to bulk sulfide phase formation. Several studies have looked at the formation and composition of these phases^2^,^18^. The exposure conditions in this work could allow poisoning of the anode by both mechanisms (S adsorption and nickel-sulfide formation). However, detection of atomic-scale S-adsorption is beyond the resolution and sensitivity limitations of the x-ray imaging techniques currently employed. On the other hand, bulk nickel-sulfide phases are easily observed, although their exact composition remains unknown. Therefore, nickel sulfides in general will be denoted in this work by Ni-S, with the understanding that this could include such possible forms as NiS, Ni_3_S_2_, Ni_3_S_4_, Ni_7_S_6_, etc^2^,^18^,^19^.

This study makes use of a synchrotron-based x-ray nanotomography (XNT) technique for obtaining 3-D data regarding the material microstructure and composition. Synchrotron sources are necessary to provide the photon energy, flux, and focusing optics to enable the study of dense samples with structural features on the nanometer to micron scale. The technique has gained popularity in recent years due to its elemental sensitivity, non-destructive nature, minimal sample preparation procedures, and developing *in-situ* imaging capabilities. A number of studies have been performed to date using XNT to examine SOFC components, both on the anode and cathode side^20^,^21^,^22^. Advances in the design of heater components and equipment has led to initial studies performing *in-situ* imaging of fuel cell components in controlled environments, and holds promise for continued future advances in the characterization of these types of materials^23^,^24^. A recent comparative study was performed by Nelson et al. on an SOFC cathode cermet of Lanthanum Strontium Manganite-Yttria Stabilized Zirconia (LSM-YSZ) using both XNT and focused ion beam-scanning electron microscopy (FIB-SEM) serial sectioning techniques to characterize the microstructure^25^. Similar results were obtained for both methods with spatial resolution of 30 nm. In its own right, FIB-SEM serial-section imaging remains a popular 3-D technique for SOFC material characterization, partially due to its greater accessibility than synchrotron experiments^26^,^27^,^28^,^29^,^30^. An extensive review of imaging techniques including XNT and FIB-SEM, specifically as applied to energy materials, was recently performed by Cocco et al^31^. Comparable imaging volumes, spatial resolution, and elemental sensitivity are typically obtained using the two methods, but XNT has been employed in this work due to its non-destructive nature as well its developing *in-situ* imaging capabilities. The non-destructive method permits ex-situ sample treatment as well as future analysis with complimentary techniques (as will be demonstrated in this work), and it is the hope of the authors that in the near future coupled 3-D chemical-morphological processes such as Ni sulfidation may be observable *in-situ*.

## Results

### Nanotomography

**Figure 1:**
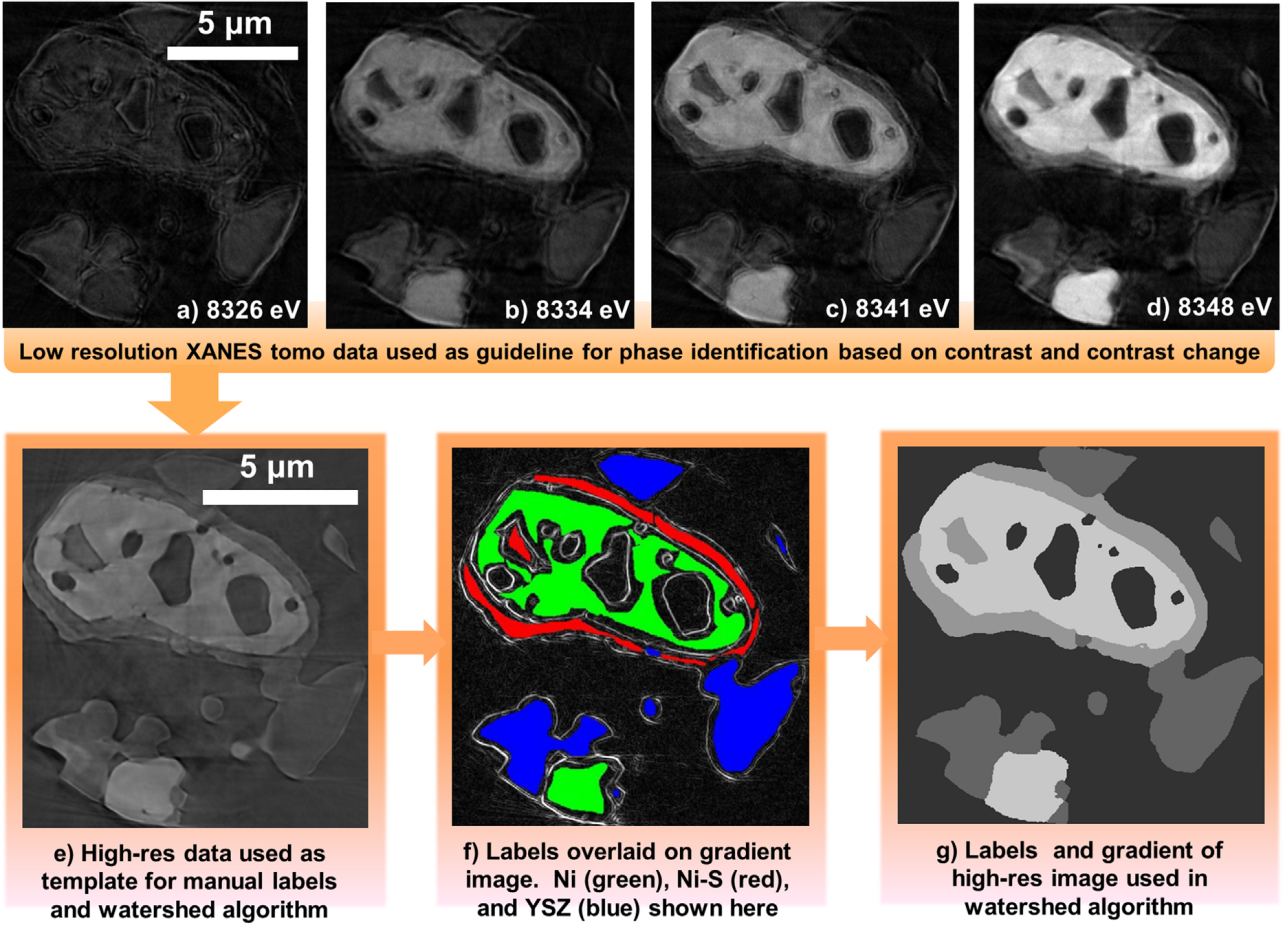
X-ray nanotomography image processing. In (a) – (d) Multi-energy tomography is performed by imaging the sample at four energy levels across the Ni k-edge. Contrast change indicates the presence of Ni, and therefore can identify Ni and Ni-S phases. In (e) a high-resolution tomography at 8350 eV with improved optics provides clear definition of edges and particle boundaries. In (f), phases were labeled based on contrast change and overlaid on the gradient of the high-resolution data. The labels were used as seed regions in a watershed algorithm, creating a segmented structure (g).

A Ni-YSZ anode was exposed to 100 ppm H_2_S in H_2_ at 800°C followed by a ramp-down to room temperature in the same gas environment. Although somewhat higher than typical H_2_S concentrations in an operating cell (on the single ppm level), 100 ppm was chosen to promote alteration of the Ni-YSZ structure in an experimentally reasonable time, and to increase the likelihood of observing the poisoning phenomena, even if done at an exaggerated level. A cylindrical sample was extracted from the anode using a FIB-SEM milling procedure and mounted for synchrotron-based x-ray nanotomography^32^. Details of the nanotomography method can be found in the methods and supplementary sections. Tomographic data was collected at 4 energy levels spanning the Ni k-edge, which were selected based on previous studies utilizing X-ray absorption near edge spectroscopy (XANES) to identify nickel and nickel sulfides^33^. The tomographic imaging of the sample used in this study was performed on the same microscope immediately following the collection of the XANES data in reference 33, ensuring consistent calibration of the energy levels. Reconstruction of the tomography projections provided 3-D structural data at each energy level. Corresponding cross sections of each data set are shown in Figure 1a–d. Nickel particles demonstrate a drastic increase in x-ray attenuation, as indicated by the bright pixels, when imaging above the k-edge at 8348 eV. On the surface of the nickel there also appears to be a scale layer which displays some increase in attenuation across the edge. At this point, this layer can be preliminarily identified as Ni-S, and will be discussed further later. The images also reveal YSZ particles, which appear as dark gray without any change across the Ni k-edge, and pore space, which is the uniform dark background.

The tomography data exhibited some noise and imaging artifacts, attributable to the beamline optics and imperfect x-ray beam focusing/alignment. Such imperfections in x-ray tomography data are typical, and are a consistent challenge with a system using x-ray focusing optics. However, since the acquisition of the original data set, recent progress in the development of zone plate lenses enabled improved imaging at better than 20 nm resolution^34^. A fifth tomographic scan was performed on the same sample at 8350 eV with the new zone plate, and significant improvements in the image quality and clarity can be observed (Fig. 1e).

Experimental time constraints did not permit data acquisition at multiple energy levels using the improved zone plate, as each tomographic scan required a run time of approximately two hours. However, identification of the different phases and segmentation of the data could be achieved using a combination of the original low-resolution data sets and the improved high-resolution data. The original data provided contrast information, allowing for approximate identification of phases, but was noisy and contained some imaging artifacts. The high-resolution data provided much clearer definition of edges and particle boundaries. To work with both together, the high-resolution data was first resized, and spatially aligned to the low-resolution data. A complete description of the subsequent labeling and segmentation process is discussed in the supplementary material, but a brief description of the workflow is provided here. A semi-manual approach was used to label regions that could be positively identified, based on contrast changes described previously, as belonging to a certain phase of material (Fig. 1f). Although only a 2-D image is shown, interpolation was used between cross-sectional slices to obtain labels for the 3-D structure. The labels were overlaid on the gradient of the high-resolution data. Taking the gradient of the data provides identification of edges and boundaries, and can be seen under the labels in Figure 1f. The labels and edges were provided as inputs to a watershed algorithm to segment the structure. The watershed is a flooding-type algorithm which iteratively “grows” the user-defined labels to label the entire structure, with constraint of the growth process dictated by the gradient edge map. Upon observation of the watershed results (Fig. 1g), manual adjustments were made to the input labels to improve some regions, such as those of low-contrast or brightness, which were not adequately captured by the watershed “flooding” process. On the second iteration of labeling, a satisfactory segmentation result was obtained with good visual agreement to the raw grayscale data.

### Fluorescence Imaging

The scale of material coating the nickel in the tomography data has, to this point, been assumed to be a nickel sulfide. While the XANES spectra of Ni and Ni_3_S_2_ were investigated previously, and used as guidelines for imaging in the current work, noise and imaging artifacts in the multi-energy tomography data (Fig. 1a–d) made it difficult to obtain exact matches between certain regions in the structure and the known reference spectra^33^. To confirm that this scale is in fact a nickel sulfide, x-ray fluorescence (XRF) spectroscopy was used to provide elemental mapping of the sample from a single orientation. Although only capable of imaging in 2-D, XRF can provide a map of the relative quantity and spatial distribution of different elements based on the emission of characteristic x-ray radiation as an x-ray spot is rastered across the sample.

**Figure 2:**
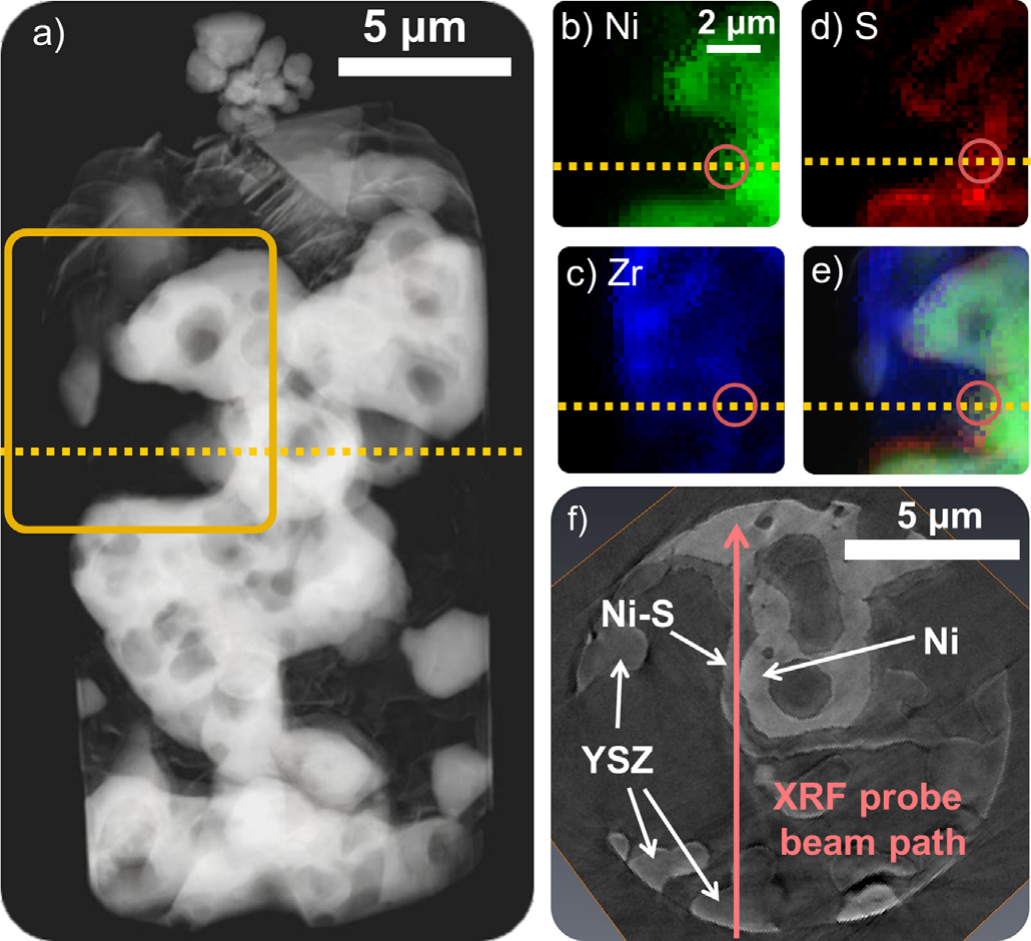
X-ray fluorescence spectroscopy of the H_2_S-treated Ni-YSZ sample. An absorption-based image (a) from the XNT experiments is compared with XRF maps of Ni (b), Zr (c), S (d). A composite map of the three elements overlaid on the corresponding region of the absorption image is also shown (e). The S signal is strongest near the edges of the Ni signal. The yellow dotted line shows the location of a cross section, extracted from the high-resolution XNT tomography, shown in (f). The pink circles in (b) – (e) and the pink arrow in (f) denote the XRF beam location and path at one particular scanning spot on the sample. The high Ni and S signals seen in (b) and (d) are correlated to a significant thickness of the scale, shown by the pink arrow in (f). The correlation of these images provides strong confidence in the identification of the scale as a nickel-sulfur compound, denoted generally by Ni-S.

Results of the XRF chemical mapping have been compared to the tomography data in Figure 2. Figure 2a is a transmission image of the entire sample taken from the tomography data. The yellow box surrounds a region used for XRF mapping (Fig. 2b–e). Figures 2b–d represent the XRF signals for Ni, Zr, and S, respectively. Note the sulfur signal (Fig. 2d) is clearly evident, and approximately correlated with the Ni signal, particularly around the edges of the Ni. In Figures 2a–e, a horizontal yellow dotted line is drawn to indicate the location from which a cross-sectional slice of the high-resolution tomography is extracted, and shown in Figure 2f. In addition, Figures 2b–e each have a pink circle, indicating one particular spot at which the XRF beam was used to acquire the Ni, Zr, and S signals. Note that the incident x-ray beam will travel through the sample, creating fluorescent x-rays throughout the thickness of the sample. At the location of this circle, the XRF indicates the presence of all three elements, including a relatively high amount of S. To correlate to the tomography data, the XRF beam path corresponding to the circular marks of Figures 2b–e has been labeled by a pink arrow in Figure 2f. The beam path clearly intercepts a significant amount of the scale, as well as some Ni and some YSZ. Because the XRF data displays a significant concentration of S at this location, and because the tomography data showed an increase in x-ray attenuation at this region when increasing x-ray energy across the Ni k-edge, it is reasonable to conclude that this scale contains both S and Ni, and is hence labeled generally as Ni-S. This validates the earlier assumption of the Ni-S scale.

## Discussion

**Figure 3:**
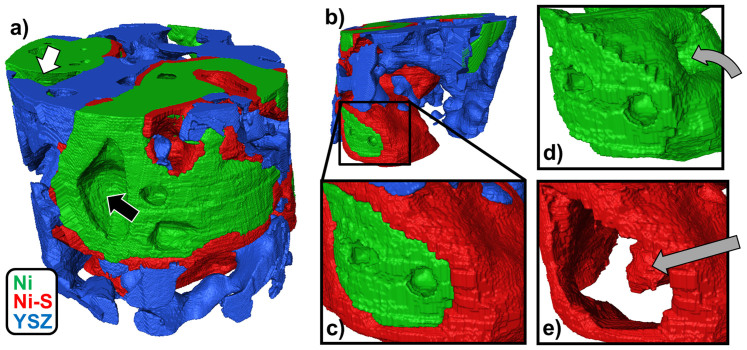
3-D renderings of the microstructure. The 3-D structure of the cylindrical sample is shown in (a). The diameter of the cylinder is about 12 microns. Recall the cylinder was milled from bulk material using FIB-SEM, so the exterior surfaces of the sample are not actually exposed surfaces. A second view is presented in (b), and a magnified picture of a particular region is shown in (c). The region shown in (c) is also used to show the distributions of the Ni phase (d) and the Ni-S phase (e).

The 3-D structure, segmented and labeled by combining information from nanotomography and XRF measurements, is shown in Figure 3. This cylindrical sample was milled from bulk material using FIB-SEM, and therefore the surfaces on the exterior of the cylinder are not true exposed surfaces. The 3-D rendering permits some qualitative observations. The Ni-S scale, shown in red, appears nearly uniformly on the Ni surface, except for in regions where Ni and YSZ are in direct contact, or areas of internal porosity, such as the large pore visible in the middle of Figure 3a labeled by the black arrow. But in addition, there are some anomalous regions of exposed Ni surface without a Ni-S scale. Such a surface can be seen in the top left of Figure 3a labeled by the white arrow. A reasonable first guess to explain this would be that the pore volume next to this Ni grain is disconnected from the other pores, and was therefore never exposed to H_2_S gas. However, more detailed examination of the internal pore structure of the sample reveals this is not the case. Ni surfaces that are both exposed and covered by a Ni-S scale are in contact with a common pore network, and have therefore been exposed to the same gas environment. (Although this is not easily discernible from the figures in this paper, the authors have used digital 3-D renderings and cross-sectional views of the internal structure to confirm the conclusion.) Presumably, this pore network within this relatively small sample is also connected to a larger pore network contiguous throughout the entire anode, as evidenced by the fact that hydrogen sulfide gas seems to have clearly penetrated the sample, creating the sulfide scale on most exposed Ni surfaces. So why is there no Ni-S scale on some Ni surfaces? Although the reason is unknown, and would require further investigation, the authors present two possibilities at this time. One possibility is that during the fabrication of the cylindrical sample by FIB milling, the porous nature of the material may have allowed the ion beam to accidentally mill some of the interior of the sample. If that is the case, it could have removed some material, possibly including Ni-S. Of the regions of exposed Ni surface, most are located near the exterior of the cylinder, meaning they could have been exposed to the ion beam. However, the exposed surfaces also exist in several locations and have faces oriented in a number of different directions, which complicates the explanation since the ion beam mills by a line-of-sight mechanism. A second possible explanation is preferential nucleation of Ni-S formation at certain locations on the Ni surface. If that were the case, it could be possible that S atoms adsorbed on the Ni surface diffused to a nearby Ni region where Ni-S formation was occurring, leaving the remaining surface exposed to the gas without formation of Ni-S. To validate this hypothesis, it would be necessary to characterize the surface orientation and surface defects of the Ni region without the Ni-S formation as well as the interface between Ni metal and Ni-S using transmission electron microscopy to reveal the surface characteristics that prevent Ni-S formation. This analysis may provide further insight into rational design of sulfur-tolerant Ni surfaces.

Qualitative observation also suggests that the Ni-S scale forms by outward diffusion of Ni ions through the sulfide scale to the gas interface (as opposed to inward diffusion of S to the Ni interface), as suggested in the literature^18^,^19^,^35^. This is apparent because of large regions of Ni-S which seem to extend into the interior of Ni particles. This effect is depicted in Figures 3b–e, which display a second angle of the microstructure. Figures 3b–c show a magnified view of one region of Ni-S scale growth. In Figure 3d, only Ni is displayed, and there is a visible cavity extending into the interior of the Ni particle, as labeled by the curved gray arrow. In Figure 3e, just the Ni-S is shown. The straight gray arrow points to a volume of Ni-S which occupies the entire cavity. It is unlikely this type of Ni-S structure would form by an inward S diffusion mechanism, as it would imply a larger S diffusion coefficient, and thus a deeper reaction penetration, in some regions of Ni versus others. More likely, these interior regions were pores before H_2_S exposure, and upon sulfidation the scale grew thicker and thicker on the interior surfaces of the Ni cavity until the entire cavity was filled by Ni-S. This explanation is consistent with previous studies which used inert markers and optical microscopy to show scale growth outward from the metal surface^36^,^37^.

Such outward scale growth is also relevant to the role of YSZ in the system. It is clear there are regions of Ni-S scale which cover Ni surfaces only to the extent at which they are exposed to pore and not in contact with YSZ. That is, there is not a Ni-S scale between Ni and YSZ. Furthermore, there is no S scale on YSZ alone, and the XRF data does not display any significant correlation between the locations of Zr and S. Therefore, it is concluded that YSZ does not participate in any chemical reaction, and serves only as a physical barrier to the growth of the Ni-S scale on Ni surfaces.

The segmented 3-D microstructure was also characterized quantitatively^38^,^39^. A voxel counting scheme was used to compute the volume fraction of each of the four phases (the three solid phases plus pore). The Ni-S scale was found to account for close to 11% of the sample volume, on the same order of magnitude as the Ni and YSZ phases, which contributed to 26 and 19 percent, respectively^39^. This ratio of total Ni to YSZ is in approximate agreement with the 55:45 weight ratio of NiO to YSZ in the starting powders of anode fabrication. Interfacial areas between the phases were also determined by a counting scheme. In the calculation of interfacial areas, isolated and internal pores were neglected. Such pores, several of which can be observed internal to Ni in the center of Figure 3a, were discounted because they are non-contributing in terms of transport and chemical/electrochemical reactive area. Considering the remaining Ni surfaces, 74% are in contact with Ni-S, 18% with YSZ, and only 8% remain exposed to pore (for unknown reasons as discussed previously). Evidently, the vast majority of what were originally clean Ni surfaces are now covered by the Ni-S scale. If the Ni-S is insulating and non-catalytic, this could have serious implications for the possible use of Ni surfaces as catalyst sites in hydrogen oxidation or hydrocarbon reformation reactions, as well as the triple phase boundary (TPB). The TPB density of the structure was calculated by a similar counting scheme, and compared to that of an “ideal” clean structure created by digitally removing Ni-S from the 3-D volume and replacing it with pore. Although only an approximation, it was found that the Ni-S formation decreases the TPB density of the anode by 67%. In the poisoned structure, only the anomalous exposed Ni surfaces, as described earlier, are still contributing to TPB density while the majority of the Ni surfaces are covered with Ni-S thus eliminating their TPB sites. This decrease in TPB sites could create a corresponding decrease in electrochemical reactions and cell performance.

**Figure 4:**
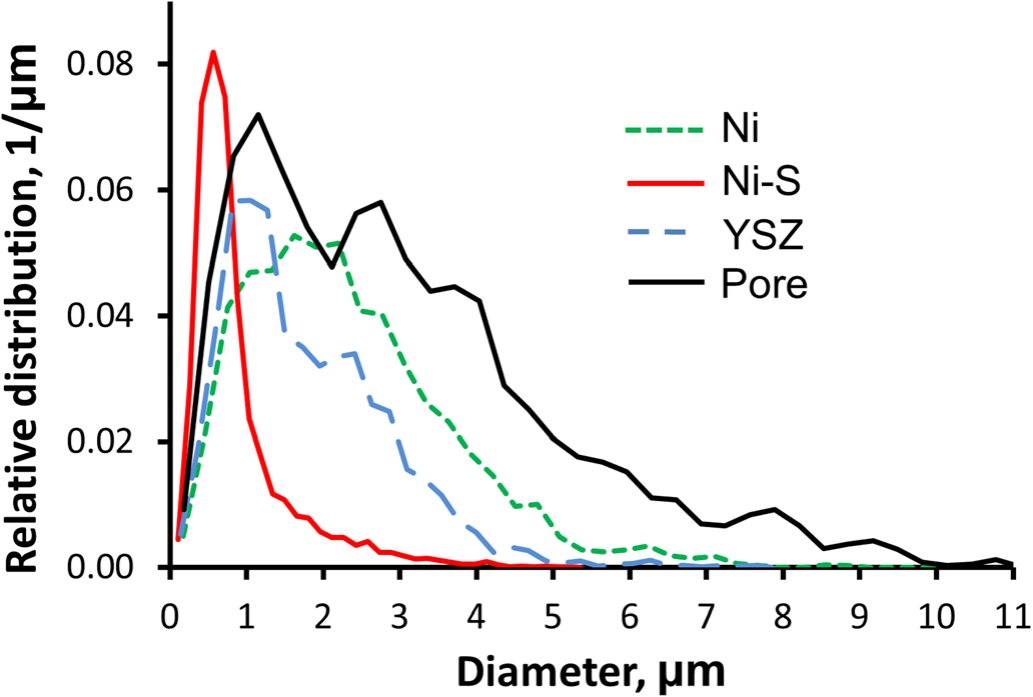
Feature sizes computed using a ray shooting algorithm. The narrow Ni-S distribution reveals that the Ni-S scale is fairly uniform, and on the order of several hundred nanometers to 1 micron thick.

To further characterize the Ni-S scale, a ray-shooting method was used to measure feature sizes of each phase^39^. The ray shooting algorithm operates separately on each phase of the sample by taking each interface point of that phase, and launching a ray into the interior of the phase in the normal direction. The ray is propagated until it hits another interface or the boundary of the domain. The lengths of all such rays are catalogued and used to form a distribution, as shown in Figure 4. Note the relative size of the sample features compared to the total sample size is important in understanding the distributions. In this sample, the Ni, YSZ, and pore phases have mostly large features close to the size scale of the entire sample (see Figure 3a). Consequently, because the sample was extracted from the bulk using FIB milling, a significant number of rays for these phases would terminate prematurely on the sample boundary, rather than continuing to propagate until another interface is reached. Therefore, the distribution represents an under-estimation of the true feature sizes, and is best interpreted as describing the relative feature sizes of the Ni, YSZ, and pore phases. The Ni-S scale, on the other hand, has generally small features with most rays traversing its thickness and terminating at the opposing interface within the structure, rather than on the boundary. Therefore, for the Ni-S phase interpretation of the quantitative results shown in the distribution is appropriate. And, as expected, the distribution of Ni-S is narrow, contained mostly within 1 micron, and is indicative of a scale of nearly uniform thickness. The peak of the distribution is located at about 570 nm. The tail on the right side of the Ni-S distribution can be attributed to locally larger regions of Ni-S, such as where it has completely filled cavities within the Ni (discussed previously), and the effect of a small number of rays that could be launched through the scale with a nearly tangent orientation, rather than in the more typical direction normal to the scale. The distributions of YSZ and Ni are quite similar to each other, and bearing in mind the discussion above, generally have feature sizes in the single micron range. The pore distribution is somewhat wider and contains a number of longer rays, covering a range of sizes from sub-micron up to the maximum measureable size, which is that of the sample itself.

In this work, the combination of x-ray nanotomography and x-ray fluorescence spectroscopy enabled the 3-D mapping of a Ni-YSZ anode poisoned by 100 ppm H_2_S. XRF was used to confirm the presence of S, and determine that its location approximately correlated with the edges of metallic Ni that were exposed to open pores. The XRF data was used to aid in the analysis of nanotomography data which was collected at several energy levels around the Ni k-edge. Data acquisition across the k-edge provided absorption contrast for Ni and a nickel/sulfur phase, denoted Ni-S for simplicity. Although the exact composition of Ni-S was not determined, its implications for electrochemical activity of the electrode could be approximated based on its spatial mapping relative to Ni, YSZ, and pore phases. The Ni-S, which formed as a nearly-uniform scale on the Ni surface, significantly decreases the amount of clean Ni surface as well as the electrochemically active triple phase boundary. The exposure conditions, which included a ramped cool-down from elevated temperature, precluded the ability to provide a detailed description of the sulfidation reaction kinetics. It is likely that the formation of the bulk Ni-S phase occurred at least in part during the ramp-down process, as opposed to the 1 hour high-temperature hold period^17^. At this point it is difficult to determine whether the reactive species creating the Ni-S was gaseous H_2_S, or atomic S adsorbed on the Ni surface during the hold period. A more rigorous and controlled experimental design would be necessary to reveal more information about the reaction mechanism. However the nature of the Ni-S formation, as a scale nearly covering the exposed Ni surface, has been presented and the characterization of the poisoned structure suggests significant implications for electrode operation after brief exposure to relatively high levels of hydrogen sulfide.

## Methods

### Sample Preparation

Ni-YSZ anodes were fabricated by a procedure similar to that reported by Yang *et al.*^40^ (but without the addition of barium oxide). Corn starch was used as a pore former to enhance porosity. The anode was part of a whole cell, which was mounted on an alumina support tube. After heated to 800 °C in air, the anode side was purged using nitrogen gas for 30 minutes before exposure to pure hydrogen to reduce the anode to the Ni-YSZ form. The fuel was then switched to H_2_ containing100 ppm H_2_S and held at 800°C for 1 hour. The cell was then cooled to room temperature at a rate of 1.5°C per minute, maintaining the 100 ppm H_2_S gas stream.

A sample for x-ray nanotomography measurements was created using a focused ion beam-scanning electron microscope (FIB-SEM). The procedure is described in detail elsewhere^32^. A cylindrical sample approximately 12 microns in diameter and 20 microns tall was milled from the bulk material using the ion beam. A micromanipulator probe was used to lift out the sample, which was then mounted to a steel pin by platinum deposition. To simplify later image processing and alignment steps, several gold particles were manually placed on the top of the cylinder with the aid of an optical microscope.

### X-ray Nanotomography

X-ray nanotomography was performed on the transmission x-ray microscope (TXM) at the Advanced Photon Source, beamline 32-ID-C. A schematic of the TXM instrumentation can be found in a referenced paper by Grew *et al*^20^. The TXM is a full-field imaging microscope, providing transmission-based images, or projections, of a sample using x-ray focusing optics, a mobile sample stage, and appropriate detector.

The synchrotron source provides a tunable incident beam, and rotation of the sample permits tomographic data collection to analyze the sample's internal 3-D structure. Tomography was initially performed 4 times on the sample using an incident x-ray beam at 4 energy levels (8326, 8334, 8341, and 8348 eV) spanning the Ni k-edge. These energy levels were chosen based on the relative contrast observed for Ni and Ni-S in previous studies utilizing X-ray absorption near edge spectroscopy (XANES)^33^,^41^. When the incident beam energy is adjusted from below to above the Ni k edge, compounds containing Ni will display a drastic increase in absorption behavior as the increased energy of the incident photons is now sufficient to provide interactions with the k-shell orbitals of the Ni atoms. This behavior is somewhat different for pure Ni as opposed to a Ni compound such as a nickel sulfide, and therefore this multi-energy imaging around the Ni k-edge was used to analyze the distribution of both Ni and Ni-S species. Ideally, imaging would also be performed across an absorption edge of sulfur, however the synchrotron beamline's monochromator and optics are not designed to directly access any of the sulfur absorption edges. For the tomographic scan at each energy level, 361 projections were acquired at half degree increments over a 180 degree range. The microscope was configured with a zoneplate lens providing 30 nm spatial resolution and a 25 nm pixel size. The projection images were then manually corrected to a central axis of rotation, and each tomographic data set was reconstructed using a filtered back projection algorithm to obtain the 3-D structure. Although a number of reconstruction algorithms exist, filtered-back projection remains a popular choice for its robustness and ease of implementation^42^.

The high resolution scan was later performed with a new zoneplate capable of providing images at better than 20 nm resolution with a pixel size of 17 nm^34^. Tomography was performed at 8350 eV by acquiring 721 images at quarter degree increments over a 180 degree range. Reconstruction was performed using a Fourier transform (FT)-based *gridrec* method, which utilizes prolate spheroidal wave functions to interpolate polar FT data onto a Cartesian grid and can achieve higher accuracy than filtered back projection^43^. The higher resolution of the raw images provided the justification for the more complex reconstruction method, to produce the best possible reconstructed data.

### X-ray Fluorescence

Synchrotron XRF was performed at the Advanced Photon Source, beamline 2-ID-D, using a focused beam with a spot size of 200 nm. XRF is used to confirm the presence of sulfur in the scale material coating the Ni surface. Because the scale is on the order of several hundred nanometers to single micron in thickness, the 200 nm spot size of the XRF probe is reasonable for obtaining a significant fluorescence signal from the elements in the scale. The resolution of the XRF is coarser than that of the tomography, the latter of which requires a small resolution to provide an adequate structural map of the morphological features of the sample. However, comparison of the XRF and tomography data is visual (as shown in Figure 2), and not on a direct point-to-point basis, so having the same resolution or pixel size for the two methods is not necessary.

General information about x-ray fluorescence measurements can be found in the literature^44^. In contrast to the full-field capabilities of the TXM, the XRF method utilizes a focused beam which is rastered across the sample to simultaneously collect spatially-resolved fluorescence signals from nickel, sulfur, and zirconium and provide a map of the Ni, Ni-S, and YSZ phases respectively. The result is a 2D elemental map of the sample. To obtain signal, the sample is exposed to a beam with a sufficiently high energy to excite electron shell transitions within the constituent atoms, the K transitions being the most prominent, followed by the lesser L and M transitions. Therefore, the detected signal contains emitted fluorescent x-rays of characteristic energy which can be prescribed to known transitions within the expected elements of Ni, S, and Zr, providing mapping capability. Note however, that for a given raster spot location, it is common to detect signal from more than one element, since the incident beam travels through the thickness of the sample, creating fluorescent signal in the interior of the sample as it progresses. Therefore, the detected signal at a given spot provides a measure of the relative amounts of the different elements along that spot's beam path. In addition, there are considerations of detector placement relative to the incident beam. Regions of the sample close to or facing the detector will provide more usable signal than regions opposite the detector, the fluorescent signal of which must travel through the sample material itself in order to reach the detector. Rotation of the sample or detector followed by an additional scan could resolve this issue if necessary, but was not done in this work.

## Author Contributions

W.M.H. and W.K.S.C. planned and designed the experiment. J.L. prepared the FIB-produced samples and obtained SEM images. W.M.H., S.W. and J. V-C. performed the nanotomography measurements. W.M.H. and G.N. performed the data processing. B.L. performed x-ray fluorescence measurements. M.L. and M.L. provided the sulfur-exposed anode sample. All authors contributed to the writing of the manuscript.

## Supplementary Material

Supplementary Information Semi-Manual Segmentation Process
