# Supplementary material for: Three-Dimensional Microstructural Imaging of Sulfur Poisoning-Induced Degradation in a Ni-YSZ Anode of Solid Oxide Fuel Cells
Source: Sci Rep. 2014 Jun 10;4:5246. doi: 10.1038/srep05246 (PMC4050380; doi:10.1038/srep05246)
Supplement: Supplementary Information — Semi-Manual Segmentation Process [file srep05246-s1.pdf]

Supplemental Section: Semi-Manual Segmentation Process

In this work, data was combined from X-ray fluorescence and a series of low-resolution tomography scans and one high-resolution scan to determine the distribution of solid and pore phases within the structure. The low-resolution scans, which were acquired using 4 x-ray energy levels spanning the Ni k-edge, provided chemical mapping information due to the change of absorption of Ni-containing species across the k-edge. Pure Ni displayed a drastic increase in its absorption when moving from below (8326 eV) to above (8348 eV) the edge, Ni-S displayed a moderate absorption increase, and the YSZ and pore phases appear the same at all energy levels. XRF measurements were used to support the conclusion of the presence of an Ni-S phase. These traits could be used to identify the 4 phases of the sample, but segmentation (also called "labeling") of the structure based on these initial low-resolution scans was still difficult. Although the phases were easily discernible by eye, the experimental noise and imaging artifacts made it impossible to digitally segment the structure using conventional methods, such as by setting threshold values for each phase.

This necessitated the use of an additional high-resolution scan to more clearly define the structure. The tomography with the improved zone plate lens provided higher image quality and lower amounts of noise and artifacts than the original scans. Interfaces between the different phases, manifested as sharp edges in the images, were much more visible in the high-resolution scan. However, because the high-resolution scan was performed at a single energy level above the Ni k-edge, it did not contain the same contrast information as the multiple-energy, low-resolution scans. Therefore, information from both data sets was combined to achieve accurate segmentation of phases.

A segmentation approach using the watershed function in MATLAB was adopted. The MATLAB watershed function uses an algorithm taken from the literature, and additional information particularly with regards to image segmentation can be found in an online article by MathWorks, the owner of MATLAB[1, 2]. The watershed algorithm is a "flooding"-type method, which takes seed regions, or "catchment basins", and iteratively expands them until the entire structure is labeled. The expansion of the seeds is guided by the edges in the image, which can be defined by taking the gradient magnitude of the image. Therefore, the expansion of a seed is restricted to cover pixels which are similar to the seed itself, and therefore all belong to the same phase of material. Of course, in an ideal world, an entire 3-D structure could be completely manually labeled as Ni, Ni-S, YSZ, or pore and there would be no need for any type of segmentation algorithm. However, such a process would be incredibly labor intensive for a structure of any appreciable size, so the watershed algorithm is implemented to decrease the total amount of manual interaction by automating part of the labeling process.

The seeds of the watershed serve as the origin of the "flooding" process, so they must represent regions which are known with certainty to belong to a certain phase. For example, if we can determine a large region of an image that is clearly the Ni phase, we would manually label that region as a Ni seed. There might be some regions which appear to be possibly Ni, but if this region is for example near an interface with another material, it may not be entirely clear whether that region belongs to the Ni phase or the other phase. Therefore, we would leave this region of the material unlabeled. This same approach is applied to the Ni, Ni-S, YSZ, and pore phases. The low-resolution tomography data sets are used to decide which parts of the structure

can be prescribed with certainty to a given phase, based on their absorption contrast change across the Ni k-edge.

The manual labeling of watershed seed regions, as described above, was performed using the "Segmentation Editor" plugin for the free software ImageJ. The 3-D data from the high-resolution scan was loaded into ImageJ as a series of 2-D cross-sectional slices. Labeling of the seed regions for each phase was done manually, using freehand-selection operations. (Throughout the process, visual reference was made to the low-resolution data sets to incorporate absorption contrast information to identify the various phases.) However, with over 300 slices of data, manually labeling every slice was not practical. Instead, labels were just provided periodically for select slices, as demonstrated in Figure S1(a). Note slices (i) and (iii) were manually labeled, shown by the green highlights, while 1 or more slices between them exemplified by (ii) are "skipped" and not labeled. The labels for these skipped slices (ii) were then created automatically by interpolating the labels from the manual slices (i) and (iii). Results of the interpolation are shown in Figure S1(b,ii). The interpolated labels frequently required manual corrections and modifications to be sure they were correct. Also, in some cases when the interpolation was not sufficiently accurate, additional slices had to be manually labeled and the interpolation repeated, in order to decrease the number of interpolated slices between manually labeled slices. The labeling process represented in Figure S1(a-b) was iterated and adjusted until a complete set of accurate labels was obtained for the entire 3-D data set, and for each of the 4 phases. (Note, Figure S1 (a-b) depict the labeling process for just the Ni phase for the sake of clarity, but the same procedure was also performed for Ni-S, YSZ, and pore phases.)

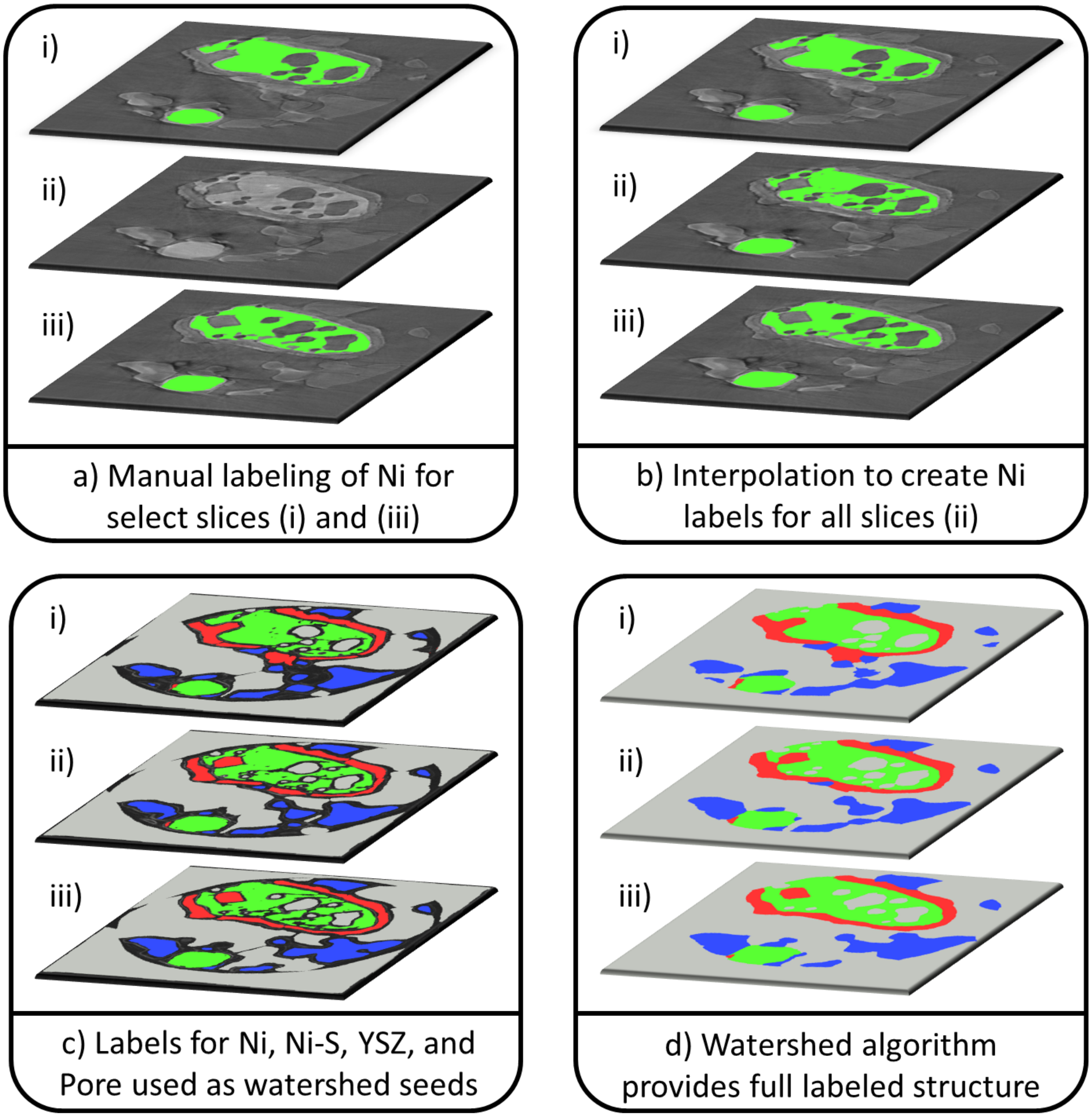

Figure S1 | Manual input to watershed algorithm. In (a), manual labeling of the 3-D data was performed for some slices, shown by (i) and (iii). Other slices (ii) were left unlabeled. An interpolation scheme was used to create labels for those slices which were not manually labeled, as shown in (b). Labels for all 4 phases (Ni, Ni-S, YSZ, and pore) for the entire volume were used as seed labels for watershed (c). Watershed segmentation provides labels for the entire structure (d).

The labels for each phase were then used as seeds in the watershed algorithm by imposing them as local minima on the gradient magnitude image. The seeds, overlaid on the gradient magnitude of the high-resolution data for visualization, are shown in Figure S1(c). Results of the watershed after the flooding process are shown in Figure S1(d). The completely labeled structure was then visually compared to the original grayscale 3-D data set to evaluate

the results (Fig. S2). This comparison was made considering the following concept: if the seed regions used as inputs to the watershed algorithm in Figure S1(c) are too sparse, the flooding process may not create a reasonable result. This is because seed regions which are surrounded by weak edges (small gradient magnitude, such as the interface between most YSZ and Ni-S regions) may flood without bound during the flooding process. Therefore, the structure would need to be more extensively labeled to better constrain the flooding process. Thus there is a trade-off: creating more seed regions requires additional manual input which is quite time consuming and labor intensive, but sufficient seed regions must be provided to sufficiently guide the watershed flooding process. For this work, the first iteration of the watershed algorithm produced some mislabeled regions. On the second iteration, additional seed regions were added as input and the results visually compared well with the grayscale data. The comparison can be seen in Figure S2, which was evaluated qualitatively to decide that the segmentation results were a reasonable representation of the grayscale structure. This "human-factor" judgment is typical in image processing and segmentation, even with simple techniques such as threshold selection methods.

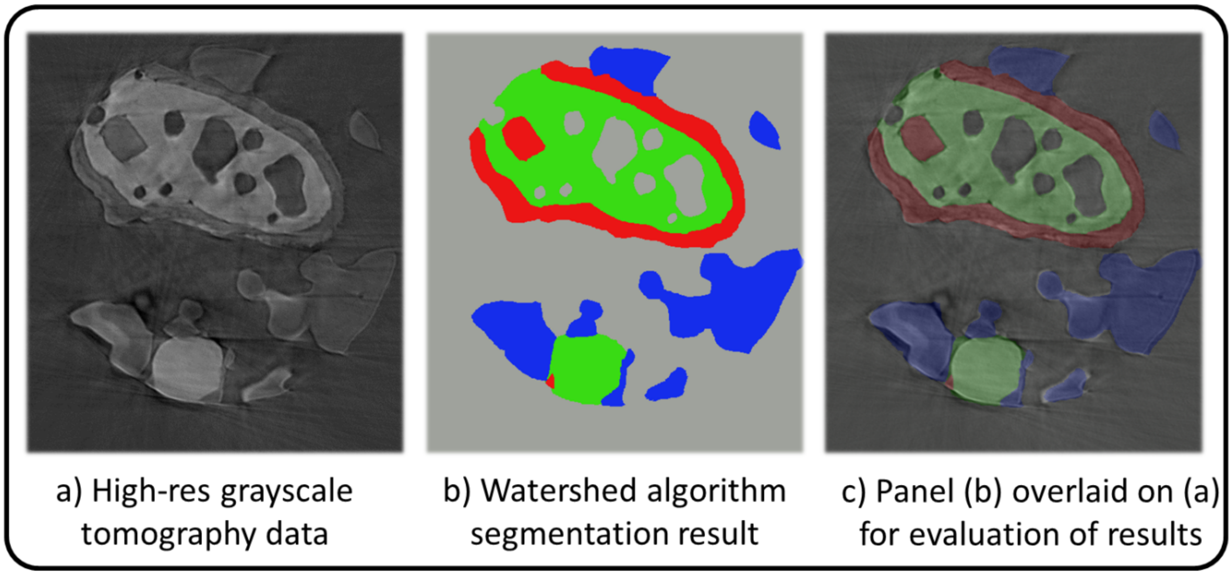

Figure S2 | Evaluation of segmentation results. One example cross-sectional slice of the original high-resolution data is shown in (a). The results of the watershed segmentation for the same cross section are shown in (b). By overlaying (b) on top of (a), we can visually assess the accuracy of the segmentation results, and determine the labels represent the structure quite well.

References

1. Meyer, F. Topographic distance and watershed lines. *Signal Process* **38**, 113-125 (1994).

2. http://www.mathworks.com/help/images/examples/marker-controlled-watershed-segmentation.html.
